# Alarming Symptoms after A Dose of Aspirin

**Published:** 1909-07-17

**Authors:** J. E. Bullock


### The General Practitioner's Column.

[Contributions to this Column are invited, and if accepted will be paid for.]

### ALARMING SYMPTOMS AFTER A DOSE OF ASPIRIN.

By J. E. BULLOCK, M.D.

About 8 p.m. the patient (male, aged 48) took a powder containing 10 grains of aspirin; within a quarter of an hour he was seized with violent itching all over the body, chiefly about the head, which he scratched vigorously. The skin felt burning hot, and tight from intense œdema; and, more especially about the face and neck, it felt drawn up into tight cords. The tongue swelled so that speech was indistinct, and there was also marked swelling of the eyelids. A sense of great oppression was felt round the throat and over the chest, the latter resembling the tightness of asthma (to which the patient is subject); he felt as if he were dying of suffocation, and he was quite unable to speak.

When a doctor arrived, in about twenty minutes, the symptoms had subsided, so that the fear of impending death was removed. The patient felt hot and was beginning to perspire, and soon most of the œdema had disappeared; the most distressing symptom then was the intense thumping of the heart, which continued several hours and prevented sleep.

When I saw the patient early next morning the only evidences of what he had gone through were some congestive blotches about the neck and an itchiness of the skin; there was no feeling of nausea, but for a day or two the appetite, which previously had been good, failed, and the bowels were unusually constipated.

The powder containing the aspirin had been obtained from a well-known and reliable chemist, and no suspicion can be attached to its composition; three powders were obtained at the same time, and one had been taken by another person with no ill effect. No alkaline medicine or mineral water was being taken by the patient. The patient states that a few years ago, after taking one tabloid (presumably 5 grains), he was seized with similar symptoms, though in a much less degree; the symptoms then resembled acute urticaria, to which the patient is subject.

Two cases have previously been recorded* in which similar symptoms occurred. In the latter case the patient had taken soda water, which is known to break up aspirin into sodium salicylate and acetate. Aspirin is insoluble in the gastric juice, and it is not until it reaches the alkaline intestinal juices that it liberates salicylic acid. The symptoms in the cases recorded occurred within a few minutes of the powder reaching the stomach, and there was no evidence of salicylism (tinnitus aurium, delirium, or gastric disturbance).

In my case the aspirin had no effect on the neuralgia for which it was given, and I can but attribute the symptoms to a peculiar idiosyncrasy of the patient, by which an immediate hyperæmia of the whole system was brought about. This is in accordance with the views of Klemperer,* who ascribes the relief given by the salicylic preparations in acute rheumatism to a general hyperæmia, especially affecting the joints. I shall be glad to hear if others have known instances of alarming symptoms after aspirin, and if any explanation can be given.

*British Medical Journal, Oct. 3 and 10, 1908. Pp. 1052 et seq., and pp. 1140 et seq.

* *Therapie der Gegenwarb*, 1907.

